# The hidden risks of polypharmacy: Exploring potentially inappropriate prescribing with STOPP/START criteria version 3–A cross-sectional study

**DOI:** 10.1371/journal.pone.0337586

**Published:** 2025-12-18

**Authors:** Mikołaj Szoszkiewicz, Ewa Deskur-Śmielecka, Arkadiusz Styszyński, Marcel Kusyń, Kamila Krzemińska, Barbara Więckowska, Agnieszka Neumann-Podczaska, Katarzyna Wieczorowska-Tobis

**Affiliations:** 1 Department of Palliative Medicine, Poznan University of Medical Sciences, Doctoral School, Geriatric Unit, Poznan, Poland; 2 Department of Palliative Medicine, Poznan University of Medical Sciences, Geriatric Unit, Poznan, Poland; 3 Department of Palliative Medicine, Poznan University of Medical Sciences, The Student Scientific Society of Poznan University of Medical Sciences, Geriatrics Research Group, Poznan, Poland; 4 Department of Computer Science and Statistics, Poznan University of Medical Sciences, Poznan, Poland; 5 University of Economics and Human Sciences in Warsaw, Senior Institute, Warsaw, Poland; Tekirdag Namik Kemal University: Tekirdag Namik Kemal Universitesi, TÜRKIYE

## Abstract

Polypharmacy and inappropriate prescribing are critical challenges in geriatric medicine, leading to adverse drug reactions, hospitalizations, and increased healthcare costs. The updated STOPP/START version 3 criteria provide an expanded tool for identifying potentially inappropriate medications (PIMs) and potentially prescribing omissions (PPOs) in older adults. This study aimed to evaluate the prevalence of PIMs and PPOs in a cohort of partially dependent older patients and identify factors associated with prescribing inappropriateness. A retrospective, cross-sectional study was conducted in two day-care centres in Poland. The study included 296 patients with polypharmacy (≥5 medications) and partial dependency. PIMs and PPOs were identified using STOPP/START version 3, and data were analyzed for factors influencing the prevalence of inappropriate prescribing. STOPP version 3 identified 543 PIMs in 73.6% of participants, with the most frequent related to analgesics as well as acetylsalicylic acid in cardiovascular indications. START version 3 detected 517 PPOs in 78.7% of patients, with cardiovascular treatments and laxatives being the most commonly omitted. Factors influencing PIMs prevalence included the number of received medications, the diagnosis of depression, and recurrent falls in the previous year. PPOs prevalence was significantly associated with multimorbidity, a high number of received medications, the diagnosis of heart failure, coronary artery disease, benign prostate hyperplasia and depression. Our study highlights the importance of using tools for optimizing pharmacotherapy due to the high prevalence of both PIMs and PPOs. Many frequent inappropriate prescribing regard relatively new medications and refer to recent updates in evidence-based medicine.

## Introduction

Polypharmacy and inappropriate treatment are pivotal geriatric problems. Polypharmacy affects a substantial proportion of the European geriatric population, ranging from 22.8% to 58.3%, depending on the country [1]. The number of prescribed drugs correlates with the probability of drug interactions, number of hospitalizations and unplanned admissions [2]. According to a systematic review containing data over a period of 10 years, the most common potentially inappropriate prescribing (PIP) were benzodiazepines, nonsteroidal anti-inflammatory drugs (NSAIDs), antihistamines and antipsychotics [3]. Thus, there is an urgent need for tools facilitating the conduct of appropriate pharmacotherapy.

STOPP/START (Screening Tool of Older Person’s Prescriptions/Screening Tool to Alert doctors to Right Treatment) criteria is a widely recognized tool developed by European geriatricians in 2008 [4]. The criteria are divided into sections, which diagnose different types of PIP: STOPP, which identifies potentially inappropriate medications (PIMs), and START, which detects potentially prescribing omissions (PPOs). In 2014 an expanded second version was published, featuring additional criteria. In 2023, the third version was released, offering 67% more drug recommendations than its predecessor [5].

The validity of previous versions has been evaluated multiple times, demonstrating a high prevalence of PIP within analysed populations [3]. Moreover, several studies assessed the impact of STOPP/START on older patient’s clinical outcomes. It turned out that the use of this tool led to a decrease in adverse drug reaction (ADR) incidence, fewer falls and lower medication costs [6,7]. The new version is a substantial extension reflecting updated literature and the registration of novel medicines. Updates reflecting the changing nature of evidence-based medicine can potentially increase their applicability in clinical practice [8]. Our preliminary analysis compared the third and second versions of the STOPP/START criteria and revealed that the new one detected more PIMs and PPOs [9]. In this study, we aimed to identify the most prevalent PIP identified by STOPP/START version 3 in a larger study group and find factors influencing their occurrence.

## Methods

The study was retrospective and cross-sectional, conducted in two day-care centres for older individuals in Poland who were partially dependent. Inclusion criteria for day-centres were: at least 60 years old, partial dependency, and hospitalization within the previous 12 months. The partial dependency was assessed using the Barthel Index, the scale used to evaluate the ability to perform activities of daily living. A score between 40 and 65 denotes partial dependency [10]. The analyses included seniors who took five or more medications daily at admission and had complete medical records. Stratified sampling was used. We analysed medical records of all patients from the day-care centre in Poznań between 2020–2023 and all patients from the day-care centre in Buk in 2023. The difference in timespan was due to two reasons: the daycare centre in Buk opened later, and there were gaps in the medical database during its initial operation.

Geriatricians conducted examinations and gathered data such as sex, age, medical history, diagnoses, biochemical test results (electrolytes, creatinine level, haemoglobin level), vaccination history (pneumococcal, COVID-19, and influenza), and drug list. Major geriatric syndromes, including depression, sleep disturbances, constipation, dementia, falls, and urinary incontinence, were actively inquired about. A geriatric team composed of a geriatrician, dietitian, psychologist, physiotherapist, occupational therapist, nurse, and social worker performed a comprehensive geriatric assessment. This study analysed an element of comprehensive geriatric assessments - Mini-Mental State Examination (MMSE). The data collected at admission were analyzed in the study. The Charlson Comorbidity Index (CCI) was estimated to evaluate the comorbidity burden. Cognitive impairment was assessed based on the MMSE, where a score below 24 or a known history of dementia indicated impairment. Data were extracted from electronic medical records between October 2023 and August 2024, with all personal identifiers removed to maintain confidentiality. Data were fully anonymised during and after analysis, thus the exemption from informed consent was approved by the Poznan University of Medical Sciences Bioethical Committee (836/22).

### Assessment of PIMs and PPOs

PIP was examined using the STOPP/START criteria version 3. The evaluation of PIMs and PPOs was performed by a resident in geriatrics, who subsequently discussed any concerns with two other geriatricians. The prevalence of PIMs and PPOs was defined as the number of patients with at least one PIM identified by STOPP version 3 (STOPP v3) or PPO identified by START version 3 (START v3). During analysis, 119 out of the 133 criteria from STOPP version 3 (89.5%) and 40 out of the 57 criteria from START version 3 (70.2%) were implemented to evaluate PIMs and PPOs.

A complete list of the STOPP/START criteria that were not implemented due to a lack of specific data can be found in our supplementary materials, S1 Table. Non-specific criteria, STOPP A1, STOPP A2 and START A1, were excluded due to vulnerability to interpretation depending on the research team. The criteria STOPP H3 (“Vitamin D supplement in older people with a confirmed 25-hydroxycholecalciferol deficiency”) were excluded due to inconsistency with national guidelines, which recommend cholecalciferol supplementation for older adults without routine screening for serum 25(OH)D [11]. On account of the lack of left ventricular ejection fraction (LVEF) value in a substantial subgroup within the study population, we excluded five additional criteria: STOPP B1 (“Digoxin for heart failure with preserved systolic ventricular function.”), START B5 (“Angiotensin Converting Enzyme inhibitors (ACE-I) for heart failure with reduced ejection fraction.”), START B6 (“Cardioselective beta-blocker for stable heart failure with reduced ejection fraction.”), START B9 (“Sacubitril/valsartan in heart failure with reduced ejection fraction causing persistent heart failure symptoms despite optimal dose of ACE-I or Angiotensin Receptor Blocker.”), START B11 (“Intravenous iron for symptomatic heart failure with reduced ejection fraction and iron deficiency.”). Criteria within the vaccine section were analyzed separately because vaccination history was available for only 82% of participants, and combining these data with other START criteria could misrepresent results due to the generally low vaccination rates for pneumococcal and influenza vaccines in the Polish population. Geriatricians actively inquired about pneumococcal and influenza vaccinations but did not ask about the varicella vaccine, leading to the exclusion of START L3 (“Varicella-zoster vaccine according to national guidelines”) from the analysis. Timespan of the study involved the year 2020 before SARS-CoV2 vaccinations, so START L4 (“SARS-CoV2 vaccine according to national guidelines”) was also excluded. Before classifying a lack of medication as a PPO, each case was carefully reviewed for possible contraindications that would qualify the medication as a PIM according to STOPP/START criteria. The full list of exclusion criteria considered is provided in the supplementary materials, S2 Table.

### Statistical analysis

Our previous research, performed in the same centres with 100 patients, served as a pilot study to calculate the sample size [9]. A proportion of 74% was used for the prevalence of PIP. A minimum sample size was estimated as 296 with a 5% significance level and a 5% margin of error. Descriptive statistics were utilized to characterize the study population and evaluate the number of PIMs and PPOs identified by STOPP/START version 3. Data was summarised as counts and percentages. Univariable logistic regression was used to evaluate the impact of the following factors on the prevalence of PIMs and PPOs: sex, age, the number of drugs, multimorbidity burden, presence of conditions prevalent among at least 10% of studied populations – diseases (arterial hypertension, heart failure (HF), coronary artery disease (CAD), atrial fibrillation (AF), anemia, diabetes mellitus type 2 (DM2), ischemic stroke, benign prostatic hyperplasia (BPH), osteoarthritis) and geriatric syndromes (urinary incontinence, depression, cognitive impairment, sleep disturbances, recurrent falls (>1 during last year), constipation). Variables with a p-value less than 0.1 were included in further multivariable analyses, each assessed individually, along with well-established factors in the literature (sex, age, and presence of cognitive impairment), to evaluate adjusted odds ratios (AOR). Logistic regression results were presented as unit odds ratios (OR) with 95% confidence intervals (CI). Based on a sample of this size, the guaranteed power of the logistic regression model would be 0.8, which would allow for detecting effect sizes (odds ratios) of at least 1.5 in a univariable model and 2 (with covariate correlations less than 0.5) in a multivariable model (G*-Power v3.1.9.6, Germany). Statistical analyses were performed using PQStat Software (2023, PQStat v.1.8.6.120, Poland), the significance level was 0.05.

## Results

### Study sample

The study sample consisted of 296 patients taking at least 5 medications. Their mean age was 79.7 years (standard deviation 7.2), ranging from 63 to 102. The majority of patients were women, representing 66% of the cohort. The CCI median score was 5 (IQR 4–6). Patients received between 5 and 22 medications, with a median of 9 (IQR: 7–11). In total, 2736 medication cases were included in the analysis.

### Potentially inappropriate prescribing

STOPP v3 identified 543 PIMs affecting 73.6% of the study cohort. The median number of PIMs per patient was 1 (IQR 0–3). According to the physiological system, the most numerous PIMs were within the central nervous system (CNS), followed by coagulation and cardiovascular systems. The most frequent PIMs included the use of regular opioids without concomitant laxatives, the use of acetylsalicylic acid (ASA) for primary prevention of cardiovascular disease, the use of drugs causing constipation in patients with chronic constipation, the use of benzodiazepines for more than 4 weeks, and the use of long-term opioids for osteoarthritis (OA). Table 1 presents a detailed list of PIMs identified by STOPP v3. Table 2 highlights the most frequent PIMs according to drug classes. Analgesics (opioids and NSAIDs) accounted for 23.4% of PIMs, whereas ASA in cardiovascular indications was responsible for 14% of PIMs. Multiple STOPP criteria regarding NSAIDs were frequent in our cohort. Among 52 patients taking NSAIDs (excluding ASA in cardiovascular doses) in our sample, 26 did not receive PPI, 21 had a history of cardiovascular disease, 9 had impaired renal function, and 6 suffered from heart failure requiring loop diuretics.

**Table 1:** Potentially inappropriate medications (PIMs) identified by STOPP version 3. Criteria with a prevalence of at least 10 patients were listed within each physiological system section along with a note of prevalence within the population.

Criteria	Number of PIMs	Percentage of population
**Central nervous system**	**100**	**33.8%**
Benzodiazepines for ≥ 4 weeks	26	8.8%
Acetylcholinesterase inhibitors with drugs that reduce heart rate	21	7.1%
Z-drugs for insomnia for > 2 weeks	13	4.4%
Drugs with potent anticholinergics/antimuscarinic effects in patients with delirium or dementia	10	3.4%
**Coagulation system**	**97**	**32.8%**
Aspirin for primary prevention of cardiovascular disease	40	13.5%
Long-term aspirin at doses greater than 100 mg per day	22	7.4%
**Cardiovascular system**	**86**	**29.1%**
Antipsychotics with a history of cardiovascular disease	21	7.1%
Non-topical NSAIDs with a history of cardiovascular disease	21	7.1%
Loop diuretics for hypertension with urinary incontinence	19	6.4%
**Falls**	**71**	**24.0%**
Antidepressants in patients with recurrent falls	19	6.4%
Antipsychotic drugs in patients with recurrent falls	11	3.7%
**Analgesics**	**46**	**15.5%**
Use of regular opioids without concomitant laxative	42	14.2%
**Musculoskeletal system**	**33**	**11.1%**
Long-term opioids for osteoarthritis	25	8.4%
**Gastrointestinal system**	**32**	**10.8%**
Drugs causing constipation in patients with constipation	32	10.8%
**Urogenital system**	**29**	**9.8%**
Systemic antimuscarinic drugs with constipation	10	3.4%
**Duplicate class**	**25**	**8.4%**
**Renal system**	**10**	**3.4%**
**Antimuscarinic**	**8**	**2.7%**
**Endocrine system**	**6**	**2.0%**
**Respiratory system**	**0**	**–**
Total	543	

NSAIDs, nonsteroidal anti-inflammatory drugs.

**Table 2:** Drug classes with the most numerous potentially inappropriate medications (PIMs) identified by STOPP version 3 within the studied population.

Group of drugs	Number of PIMs	Percentage of PIMs
Acetylsalicylic acid in cardiovascular indications	76	14%
Opioids	75	13.8%
NSAIDs	52	9.6%
Muscarinic antagonists	42	7.7%
Benzodiazepines	40	7.4%
Antipsychotics	34	6.3%
Antidepressants	27	5%
Loop diuretics	22	4.1%

NSAIDs, nonsteroidal anti-inflammatory drugs.

START v3 identified 517 PPOs affecting 78.7% of the study cohort. The median number of PPOs per patient was 2 (IQR 1–3). More than one-third (37.7%) of PPOs were within the cardiovascular system, followed by the gastrointestinal (GI) and urogenital systems. The most frequent PPOs included the lack of laxatives in patients with chronic constipation and/or receiving opioids, the lack of sodium-glucose cotransporter-2 (SGLT-2) inhibitors and mineralocorticoid receptor antagonist (MRA) in heart failure, and the lack of statins in patients with a history of cardiovascular disease. Table 3 presents a detailed list of PPOs identified by START v3. Table 4 highlights the most frequent PPOs according to drug classes. Laxatives accounted for the highest number of PPOs, followed by SGLT-2 inhibitors, and MRA. Constipation was present in 28.4% of the studied sample (84 individuals). Simultaneously, only 2.0% of patients received regular laxatives. We evaluated vaccine-related criteria for 242 patients. Among them, 2.9% of patients (7 individuals) reported pneumococcal vaccination, while 12.0% of patients (29 individuals) reported influenza vaccination.

**Table 3:** Potentially prescribing omissions (PPOs) identified by START version 3. Criteria with a prevalence of at least 10 patients were listed within each physiological system section along with a note of prevalence within the population.

Criteria	Number of PPOs	Percentage of population
**Cardiovascular system**	**195**	**65.9%**
SGLT-2 inhibitors in heart failure	62	21.0%
Mineralocorticoid receptor antagonist in heart failure	50	17.0%
Statin therapy with a history of cardiovascular disease	32	10.8%
ACE inhibitor with coronary artery disease	26	8.8%
Beta-blocker with symptomatic coronary artery disease	18	6.1%
**Gastrointestinal system**	**119**	**40.2%**
Osmotic laxative for chronic constipation	78	26.4%
Proton pump inhibitor with NSAIDs	26	8.8%
**Urogenital system**	**53**	**17.9%**
5-alpha reductase inhibitor for lower urinary tract symptoms related to benign prostatic hyperplasia	27	9.1%
Alpha-1 blocker for lower urinary tract symptoms related to benign prostatic hyperplasia	25	8.4%
**Analgesics**	**43**	**14.5%**
Laxatives in patients receiving opioids	42	14.2%
**Central nervous system**	**35**	**11.8%**
Non-TCA drug for major depression	22	7.4%
**Musculoskeletal system**	**35**	**11.8%**
Bisphosphonates or anabolic therapy for osteoporosis	15	5.1%
**Coagulation system**	**25**	**8.4%**
Antiplatelets therapy with a history of cardiovascular disease	16	5.4%
**Respiratory system**	**12**	**4.1%**
Long-acting muscarinic antagonist or long-acting beta 2 agonist for symptomatic COPD and asthma	12	4.1%
**Endocrine system**	**0**	**–**
**Renal system**	**0**	**–**
Total	517	

ACE, angiotensin-converting enzyme; SGLT-2, sodium-glucose cotransporter-2; Non-TCA, non-tricyclic antidepressants; NSAID, nonsteroidal anti-inflammatory drug.

**Table 4:** Drug classes with the most numerous potentially prescribing omissions (PPOs) identified by STOPP version 3 within the studied population.

Group of drugs	Number of PPOs	Percentage of PPOs
Laxatives	126	24.4%
SGLT-2 inhibitors	62	12.0%
Mineralocorticoid receptor antagonists	50	9.7%
Statins	32	6.2%
PPIs	31	6.0%
Antidepressants	29	5.6%
5-alpha reductase inhibitor	27	5.2%
ACE inhibitors	26	5.0%

ACE, angiotensin-converting enzyme; SGLT-2, sodium-glucose cotransporter-2; PPIs, protone pump inhibitors.

### Factors associated with potentially inappropriate prescribing

Factors mentioned in the methods section were analysed in univariable logistic regression for the prevalence of PIMs and separately the prevalence of PPOs (Tables 5 and 6). All factors with p-values <0.1 were bolded in tables. Those factors were further analysed in multivariable analysis with sex, age, and cognitive impairment. Regarding PIMs, the number of received drugs was by far the most decisive factor leading to PIMs with OR 1.26 [CI 1.13–1.4]. Apart from this, the presence of depression and recurrent falls in medical history were associated with a risk of PIMs. The most influential factors inducing PPOs were the diagnosis of HF with OR 23.99 [CI 3.26–176.6], CAD with OR 8.32 [CI 2.92–23.7], and BPH with OR 5.76 [CI 1.35–24.58]. Multimorbidity, the number of received drugs, and the diagnosis of depression also increased the risk of PPOs. Regarding presence of specific diseases, some subgroups without PIMs/PPOs included very few cases, which may bias the univariable results and particularly affect the interpretation of odds ratios. To ensure transparency, all univariable results with subgroup counts are provided in the Supplementary Materials, S3 Table. Due to the separation problem convergence criterion was not met in the case of constipation, so we could not include it in regression models. Based on descriptive statistics we assume that constipation is an important factor inducing PPOs. Among 84 patients suffering from constipation, 92.9% did not receive laxatives, and this criterion was the most frequent PPO.

**Table 5:** Univariate logistic regression analysis regarding presence of at least one potentially inappropriate medication (PIM). Factors with p-value less than 0.1 were bolded and further analysed in multivariable regression with sex, age and the presence of cognitive impairment to assess the adjusted odds ratio (AOR).

Variables	Odds ratio	−95% CI	+95% CI	p-value	Adjusted odds ratio	−95% CI	+95% CI	p-value
**The number of drugs**	**1.26**	**1.13**	**1.4**	**<0.01**	**1.26**	**1.13**	**1.40**	**<0.01**
**Comorbidity burden**	**1.15**	**0.98**	**1.36**	**0.10**	**1.15**	**0.95**	**1.39**	**0.15**
**Depression**	**2.00**	**1.01**	**3.95**	**0.048**	**2.15**	**1.05**	**4.44**	**0.04**
**Recurrent falls**	**2.54**	**1.14**	**5.64**	**0.02**	**2.40**	**1.07**	**5.38**	**0.03**
Sex	0.76	0.44	1.30	0.31				
Age	1.02	0.99	1.06	0.25				
Cognitive impairment	1.20	0.69	2.10	0.51				
Hypertension	0.85	0.38	1.89	0.69				
Heart failure	0.78	0.42	1.42	0.41				
Coronary artery disease	0.94	0.53	1.64	0.81				
Anemia	0.64	0.33	1.25	0.19				
Diabetes mellitus type 2	0.91	0.53	1.55	0.73				
Atrial fibrillation	0.62	0.35	1.12	0.11				
Benign prostate hyperplasia	0.59	0.29	1.21	0.15				
Urinary incontinence	1.54	0.91	2.61	0.11				
Ischemic stroke	1.05	0.58	1.90	0.87				
Sleep disturbances	0.91	0.44	1.88	0.80				
Constipation	1.20	0.67	2.17	0.53				
Osteoarthritis	1.18	0.68	2.05	0.56				

**Table 6:** Univariate logistic regression analysis regarding presence of at least one potentially prescribing ommision (PPO). Factors with p-value less than 0.1 were bolded and further analysed in multivariable regression with sex, age and the presence of cognitive impairment to assess the adjusted odds ratio (AOR).

Variables	Odds ratio	−95% CI	+95% CI	p-value	Adjusted odds ratio	−95% CI	+95% CI	p-value
**The number of drugs**	**1.15**	**1.04**	**1.28**	**<0.01**	**1.15**	**1.04**	**1.28**	**<0.01**
**Comorbidity burden**	**1.31**	**1.09**	**1.59**	**<0.01**	**1.43**	**1.12**	**1.8**	**<0.01**
**Depression**	**2.08**	**0.97**	**4.46**	**0.06**	**2.20**	**1.01**	**4.78**	**0.02**
**Benign prostate hyperplasia**	**5.76**	**1.35**	**24.58**	**0.02**	**8.34**	**1.81**	**38.37**	**<0.01**
**Heart failure**	**23.99**	**3.26**	**176.60**	**<0.01**	**24.1**	**3.27**	**178.05**	**<0.01**
**Coronary artery disease**	**8.32**	**2.92**	**23.70**	**<0.01**	**8.63**	**2.99**	**24.89**	**<0.01**
**Atrial fibrillation**	**2.03**	**0.95**	**4.37**	**0.07**	**1.98**	**0.91**	**4.28**	**0.08**
Sex	1.03	0.57	1.85	0.93				
Age	1.02	0.98	1.06	0.42				
Cognitive impairment	0.94	0.52	1.68	0.83				
Hypertension	1.17	0.52	2.62	0.70				
Anemia	1.38	0.61	3.13	0.44				
Diabetes mellitus type 2	1.33	0.73	2.40	0.35				
Urinary incontinence	1.41	0.80	2.48	0.24				
Ischemic stroke	1.33	0.69	2.56	0.40				
Recurrent falls	1,56	0,72	3,38	0.26				
Sleep disturbances	0,57	0,28	1,17	0.12				
Osteoarthritis	1,28	0,71	2,30	0.42				

## Discussion

Our study is one of the first to evaluate the PIP detected by STOPP/START criteria, version three. We noticed the high prevalence of PIMs identified by STOPP v3 and PPOs detected by START v3 in the studied population. A high proportion of PIMs were associated with using analgesics, mainly NSAIDs, but also opioids. A large part of PPOs was due to a lack of appropriate treatment for cardiovascular diseases and constipation. This study continues previous preliminary research, enabling the representative sample of a partially dependent geriatric population with polypharmacy [9].

NSAIDs (excluding ASA in cardiovascular doses) and opioids were responsible for 23.4% of PIMs identified by STOPP v3. NSAIDs remain one of the most popular analgesics with various indications, including OA, inflammatory diseases and various pain symptoms. The usefulness of this drug class is restricted due to various adverse effects: nausea, diarrhea, gastrointestinal bleeding, worsening renal function (including acute kidney injury), exacerbation of heart failure, and increased risk of major vascular events [12]. In the geriatric population, special precautions should be performed due to the high prevalence of comorbidity and higher risk of adverse effects. Thus, authors of STOPP/START criteria addressed multiple criteria about NSAIDs [5]. As described in the result section, most of the patients receiving NSAIDs within our study sample had relevant contraindications for them.

A substantial part of analgesic-related PIMs were due to incorrect management of OA. In our cohort, 16.1% of patients with OA were treated with NSAIDs, whereas 12% of patients with OA were treated with long-term opioid therapy (mainly tramadole). International guidelines are either conditionally for or weakly against tramadol and other opioid usage in people with OA. The authors point to opioids’ harmful effects and potential to develop dependency [13,14]. Moreover, recent meta-analyses indicate that only a limited subset of carefully selected patients with OA derive significant benefits from prolonged opioid therapy [15]. Thus, in the geriatric population, affected mainly by comorbidity, non-pharmacologic treatment should play a pivotal role. In patients with contraindications to NSAIDs, intra-articular and topical therapies should be considered [14,15].

The most common PPOs detected by START v3 were the lack of osmotic laxatives in patients with chronic constipation. Widespread constipation and almost complete lack of use of osmotic laxatives remained one of the central problems in our population. This issue becomes even more serious due to the common use of opioids in questionable indications, such as above mentioned OA. Constipation markedly decreases the quality of life, leading to both somatic (haemorrhoids, bowel obstruction) and psychiatric disorders (depression, anxiety) [16,17]. Early intervention and prophylaxis during opioid therapy are recommended for the management of constipation [18].

PPOs related to the cardiovascular system were notably common, representing 38% of all instances. Among this category, heart failure accounted for the highest number of PPOs, followed by coronary artery disease. The lack of SGLT-2 inhibitors in heart failure was particularly numerous, confirming our previous results [9]. During the last two years, European and American cardiologic associations introduced a substantial change in guidelines for the treatment of heart failure and recommended SGLT-2 inhibitors regardless of LVEF [19,20]. Recent extension of indications for SGLT-2 inhibitors may be the main reason for the frequency of this omission. The authors of STOPP/START v3 indicate the use of MRA in heart failure without reference to reduced LVEF, unlike ACE-I and beta-blockers, which include such a reference. This distinction is discordant with the updated European Society of Cardiology Guidelines. In guidelines, there are the same indications for ACE-I, beta blockers and MRA: strong recommendation (IA) for treatment in patients with HF with reduced LVEF, weak recommendation (IIb) for treatment in patients with HF with mildly reduced LVEF and no recommendation for HF with preserved LVEF. Apart from SGLT-2 inhibitors, other disease-modifying therapies did not achieve positive endpoints in clinical trials in patients with HFpEF [19]. Thus, it might be worth considering adding a reference to reduced ejection fraction for MRA in future STOPP/START criteria.

The most influential factor inducing PIMs was the number of received drugs. The connection between multiple drug use and inappropriate medication, followed by clinical consequences, is well-established in the literature [21]. More surprisingly, the number of received drugs increased (although less prominently) the risk of PPOs. Our finding corresponds with previous research describing STOPP/START v2 [22,23]. It seems that using multiple drugs does not protect against omissions and even complicates the use of appropriate medications.

The presence of heart failure and coronary artery disease were influential factors in the presence of PPOs. This derives from particularly widespread omissions regarding SGLT-2 inhibitors, MRA, beta-blockers, ACE-I and statins. Depression was associated with both the presence of PIM and PPO. The problem of polypharmacy and depression is well-described in the literature, showing a relevant association between them [24]. In our study, more than 7% of the population with major depression did not receive appropriate treatment, which may influence the regression models. Interestingly, recurrent falls were an independent factor only for the presence of PIM and not PPO. This emphasizes the impact of drugs on balance problems in older patients. A recent systematic review revealed that patients with inappropriate treatment are at higher risk of falls and fractures [25]. However, given the relatively small number of participants without PPOs, some subgroups included only very few cases, which may bias the results. In this context, the strength of association expressed as odds ratios for conditions such as heart failure and coronary artery disease should be interpreted with caution. Although sex, age, and cognitive impairment did not significantly influence PIP in our univariable analysis, we included them in the multivariable because these are well-known factors corresponding with inappropriate medication [26,27]. Therefore, we proved that comorbidity burden, the number of drugs and the presence of the conditions mentioned above influence the presence of PIM/PPO independently of those variables.

The study has some limitations. First, we assessed a selected group of geriatric population – partially dependent patients with polypharmacy. Results may not be generalisable to seniors with functional independence and those taking fewer medications. Although the study was based on a calculated sample size and stratified sampling, some estimates regarding the influence of specific comorbidities on prescribing errors should be interpreted with caution due to limited subgroup sizes. Still, polypharmacy affects more than half of Polish seniors, and we maintain that our results refer to a substantial part of the population [28]. Secondly, this is a retrospective study whose design precludes the assessment of the clinical outcomes after intervention with STOPP/START criteria. Future research should include prospective trials to evaluate clinical outcomes. Nonetheless, our study showed the high prevalence of PIP detected by the STOPP/START criteria version 3. We assessed a representative group of partially dependent seniors, and we identified the most significant issues affecting the prevalence of pharmacotherapy inappropriateness.

Our study is an influential voice in the ongoing debate about the problem of inappropriate medication. Previous versions of STOPP/START were used in many observational studies and proved to be an effective tool for searching PIP [29]. Moreover, interventional trials revealed that using STOPP/START version 2 decreases the number of adverse drug reactions as well as certain clinical outcomes like the number of falls [6,7]. The new version of the tool contains 76 more criteria and multiple relevant modifications regarding previous ones. This study was one of the first worldwide to assess the most important PIP in the geriatric population using STOPP/START v3. Many new trends in medicine, such as SGLT-2 inhibitors in heart failure or no opioids in OA, were among the most common inappropriateness. This underscores the necessity of relying on new tools whose updates respond to the evolving nature of medicine.

## Supporting information

S1 Table Not used STOPP/START criteria version 3. (DOCX)

S2 Table STOPP/START exclusion criteria applied for PPO assessment. (DOCX)

S3 Table Distribution of nominal variables including in univariate logistic regressions regarding potentially inappropriate medications. (DOCX)

S1 Data Dataset used in this study. (XLSX)
