# Supplementary material for: The hidden risks of polypharmacy: Exploring potentially inappropriate prescribing with STOPP/START criteria version 3–A cross-sectional study
Source: PLoS One. 2025 Dec 18;20(12):e0337586. doi: 10.1371/journal.pone.0337586 (PMC12714279; doi:10.1371/journal.pone.0337586)
Supplement: S1 Table — (DOCX) [file pone.0337586.s001.docx]

| **Not used STOPP/START criteria from version 3** | | | |
| --- | --- | --- | --- |
| PART | SECTION | CRITERIUM | |
| STOPP | Indication  of medication | A1 | Any drug prescribed without a clinical indication. |
|  |  | A2 | Any drug prescribed beyond the recommended duration, where treatment duration is well defined. |
|  | Cardiovascular System | B1 | Digoxin for heart failure with preserved systolic ventricular function (no clear evidence of benefit. |
|  |  | B8 | Loop diuretic for dependent ankle oedema without clinical, biochemical evidence or radiological evidence of heart failure, liver failure, nephrotic syndrome or renal failure (leg elevation and /or compression hosiery usually more appropriate). |
|  |  | B13 | Aldosterone antagonists (e.g., spironolactone, eplerenone) with concurrent potassiumconserving drugs (e.g., ACEI’s, ARB’s, amiloride, triamterene) without monitoring of serum potassium (risk of dangerous hyperkalaemia i.e., > 6.0 mmol/l – serum K should be monitored regularly, i.e., at least every 6 months). |
|  |  | B15 | Drugs that predictably prolong the QTc interval (QTc = QT/RR) in patients with known with demonstrable QTc prolongation (to >450 msec in males and >470 msec in females), including quinolones, macrolides, ondansetron, citalopram (doses > 20 mg/day), escitalopram (doses > 10 mg/day), tricyclic antidepressants, lithium, haloperidol, digoxin, class 1A antiarrhythmics, class III antiarrhythmics, tizanidine, phenothiazines, astemizole, mirabegron (risk of lifethreatening ventricular arrhythmias). |
|  |  | B16 | Statins for primary cardiovascular prevention in persons aged ≥ 85 and established frailty with expected life expectancy likely less than 3 years (lack of evidence of efficacy). |
|  | Central Nervous System | D5 | Antipsychotics prescribed for behavioural and psychological symptoms of dementia (BPSD) an unchanged dose for > 3 months without medication review (increased risk of extrapyramidal side-effects and chronic worsening of cognition, increased risk of major cardiovascular morbidity and mortality). |
|  |  | D15 | Antipsychotics in patients with behavioural and psychological symptoms of dementia (BPSD) for longer than 12 weeks unless BPSD symptoms are severe and other nonpharmacological treatments have failed (increased risk of stroke, myocardial infarction). |
|  | Musculoskeletal System | H3 | Long-term use of NSAID (>3 months) for symptom relief of osteoarthritis pain where paracetamol has not been tried (simple analgesics preferable and usually as effective for pain relief). |
|  | Urogenital System | I2 | Systemic antimuscarinic drugs in patients with narrow-angle glaucoma (risk of acute exacerbation of glaucoma). |
|  | Endocrine System | J9 | Levothyroxine in subclinical hypothyroidism i.e., normal free T4, elevated TSH but < 10 mU/L (no evidence of benefit, risk of iatrogenic thyrotoxicosis). |
|  | Analgesic Drugs | L1 | Use of oral or transdermal strong opioids (morphine, oxycodone, fentanyl, buprenorphine, diamorphine, methadone, tramadol, pethidine, pentazocine) as first line therapy for mild pain (WHO analgesic ladder not observed; paracetamol or NSAID not prescribed as first-line therapy). |
|  |  | L3 | Long-acting opioids without short-acting opioids for break-through moderate or severe pain (risk of persistence of severe pain). |
| START | Indicated drugs | A1 | Where a drug is clearly indicated and considered appropriate in the particular clinical context and there is no clear contraindication, that drug should be initiated as per formulary guidelines for dose and duration. |
|  | Cardiovascular System | B5 | Angiotensin Converting Enzyme (ACE) inhibitor for heart failure with reduced ejection fraction. |
|  |  | B6 | Cardioselective beta-blocker (bisoprolol, nebivolol, metoprolol or carvedilol) for stable heart failure with reduced ejection fraction. |
|  |  | B9 | Sacubitril/valsartan in heart failure with reduced ejection fraction causing persistent heart failure symptoms despite optimal dose of ACE inhibitor or Angiotensin Receptor Blocker (Sacubitril/valsartan to replace ACE inhibitor or Angiotensin Receptor Blocker). |
|  |  | B10 | Beta-blocker for chronic atrial fibrillation with uncontrolled heart rate. |
|  |  | B11 | Intravenous iron for symptomatic heart failure with reduced ejection fraction and iron deficiency. |
|  | Renal System | E1 | One-alpha hydroxycholecalciferol or calcitriol supplementation in severe chronic kidney (i.e., eGFR < 30 ml/min/m2) disease with hypocalcaemia (corrected serum calcium < 2.10 mmol/l) and associated secondary hyperparathyroidism. |
|  |  | E2 | Phosphate binder in severe chronic kidney disease (i.e., eGFR < 30 ml/min/m2) if serum phosphate concentration persistently >1.76 mmol/l (5.5 mg/dl) despite adherence to renal diet. |
|  |  | E4 | Angiotensin receptor blocker (ARB) or Angiotensin Converting Enzyme Inhibitor (ACE-I) in chronic kidney disease with proteinuria i.e., urine albumin excretion >300 mg/24 hours. |
|  | Respiratory System | G2 | Regular i.e. daily inhaled corticosteroid (e.g., beclomethasone, budesonide, ciclesonide, fluticasone, mometasone) for moderate-severe asthma or COPD of GOLD 3 or 4 severity, where FEV1 <50% of predicted value and repeated exacerbations requiring treatment with oral corticosteroids. |
|  | Musculoskeletal System | H5 | Vitamin D supplement in older people with confirmed 25-hydroxycolecalciferol deficiency (< 20 micrograms/L, < 50 nmol/L) who are housebound or experiencing falls or with osteopenia (Bone Mineral Density T-score is less than -1.0 but above -2.5 in one or multiple sites). |
|  |  | H6 | Anti-resorptive treatment after discontinuation of at least two doses of denosumab (rebound increased bone turnover markers, BMD loss, and increased risk of vertebral fracture following denosumab discontinuation). |
|  |  | H7 | Anti-resorptive treatment after discontinuation of teriparatide/abaloparatide treatment for osteoporosis. |
|  | Endocrine System | J1 | ACE inhibitor or Angiotensin Receptor Blocker (if intolerant of ACE inhibitor) in diabetes with evidence of renal disease i.e., dipstick proteinuria or microalbuminuria (>30 mg/24 hours) unless evidence of severe CKD (eGFR < 30 ml/min/m2). |
|  | Analgesics | K1 | High-potency opioids in moderate-severe pain, where paracetamol, NSAIDs or low-potency opioids are not appropriate to the pain severity or have been ineffective. |
|  | Vaccines | L3 | Varicella-zoster vaccine according to national guidelines. |
|  |  | L4 | SARS-CoV2 vaccine according to national guidelines |
| ACEI, ACE-I, angiotensin-converting enzyme inhibitors; ARB, angiotensin receptor blocker; QTc, QT interval corrected for heart rate; QT, QT interval; RR, interval from the onset of one QRS complex to the onset of the next QRS complex; BPSD, behavioural and psychological symptoms of dementia; WHO, World Health Organization; NSAID, nonsteroidal anti-inflammatory drug; ACE, angiotensin-converting enzyme; eGFR, estimated glomerular filtration rate; COPD, chronic obstructive pulmonary disease; GOLD, The Global Initiative for Chronic Obstructive Lung Disease; FEV1, forced expiratory volume in one second; BMD, bone mineral density. | | | |
