# Supplementary material for: The hidden risks of polypharmacy: Exploring potentially inappropriate prescribing with STOPP/START criteria version 3–A cross-sectional study
Source: PLoS One. 2025 Dec 18;20(12):e0337586. doi: 10.1371/journal.pone.0337586 (PMC12714279; doi:10.1371/journal.pone.0337586)
Supplement: S2 Table — (DOCX) [file pone.0337586.s002.docx]

Table S2: STOPP/START exclusion criteria applied for PPO assessment

| Medication class | START criteria | STOPP criteria |
| --- | --- | --- |
| Angiotensin-Converting Enzyme inhibitors (ACEIs) or Angiotensin Receptor Blockers  (ARBs) | **B.3** Angiotensin Converting Enzyme (ACE) inhibitor with coronary artery disease | **B.12** Angiotensin-Converting Enzyme inhibitors (ACEIs) or Angiotensin Receptor Blockers  (ARBs) in patients with hyperkalaemia i.e., serum K > 5.5 mmol/l |
| Beta-blockers | **B.4** Beta-blocker with symptomatic coronary artery disease | **B3.** Beta-blocker in combination with verapamil or diltiazem (risk of heart block).  **B.4** Ventricular rate-limiting drugs i.e., beta blocker, […] with bradycardia (< 50/min), type II heart block or complete heart block (risk of complete heart block, asystole).  **J.3** Non-selective beta-blockers in diabetes mellitus with frequent hypoglycaemic episodes (risk of suppressing hypoglycaemic symptoms) |
| Propranolol | **D7.** Propranolol for essential tremor with functional impairment and resultant disability. | **J3.** Non-selective beta-blockers in diabetes mellitus with frequent hypoglycaemic episodes (risk of suppressing hypoglycaemic symptoms). |
| SGLT-2 inhibitors | **B.8** SGLT-2 inhibitors (canagliflozin, dapagliflozin, empagliflozin, ertugliflozin) in symptomatic  heart failure with or without reduced ejection fraction regardless of diabetes being present  or not) | **J.4** Sodium glucose co-transporter (SGLT2) inhibitors (e.g., canagliflozin, dapagliflozin, empagliflozin, ertugliflozin) with symptomatic hypotension (risk of exacerbation of hypotension) |
| Vitamin K antagonists or direct thrombin inhibitors or factor Xa inhibitors or antiplatelets | **C.1** Vitamin K antagonists or direct thrombin inhibitors or factor Xa inhibitors in the presence  of chronic or paroxysmal atrial fibrillation  **C.2** Antiplatelet therapy (aspirin or clopidogrel or prasugrel or ticagrelor) with a documented  history of coronary, cerebral or peripheral vascular disease. | **C.2** Antiplatelet agents, vitamin K antagonists, direct thrombin inhibitors or factor Xa inhibitors with concurrent significant risk of major bleeding, i.e. uncontrolled severe hypertension, bleeding diathesis, recent non-trivial spontaneous bleeding (high risk of bleeding).  **F.6** Antiplatelet or anticoagulant drugs with a history of Gastric Antral Vascular Ectasia (GAVE, “watermelon stomach”) (risk of major gastrointestinal bleeding)  **E.3** Factor Xa inhibitors (e.g., rivaroxaban, apixaban, edoxaban) if eGFR < 15 ml/min/1.73m2  (risk of bleeding) |
| Acetylcholinesterase inhibitor | **D.3** Acetylcholinesterase inhibitor (donepezil, rivastigmine, galantamine) for mild-moderate  Alzheimer’s dementia  **D4.** Rivastigmine for Dementia with Lewy Bodies or Parkinson’s disease dementia. | **D.17** Acetylcholinesterase inhibitors with a known history of persistent bradycardia (< 60 beats/min.), heart block or recurrent unexplained syncope (risk of cardiac conduction failure, syncope and injury).  **D.18** Acetylcholinesterase inhibitors with concurrent treatment with drugs that induce persistent bradycardia (< 60 beats/min.) such as beta-blockers, digoxin, diltiazem, verapamil (risk of cardiac conduction failure, syncope and injury). |
| Bisphosphonates | **H.2** Bisphosphonates and [...] in patients taking long-term systemic  corticosteroid therapy for prevention of steroid-induced osteoporosis | **H.8** Oral bisphosphonates in patients with a current or recent history of upper gastrointestinal disease i.e. dysphagia, oesophagitis, gastritis, duodenitis, or peptic ulcer disease, or upper gastrointestinal bleeding (risk of relapse/exacerbation of oesophagitis, oesophageal ulcer, oesophageal stricture) |
| Selective alpha-1 receptor blocker | **I.1** Selective alpha-1 receptor blocker (e.g., tamsulosin, silodosin) for lower urinary tract  symptoms related to benign prostatic hyperplasia where prostatectomy is not considered  necessary or appropriate or safe | **I.5** Alpha-1 receptor antagonists other than silodosin (e.g., alfuzosin, doxazosin, indoramin, tamsulosin, terazosin) with symptomatic orthostatic hypotension or history of syncope (risk of precipitating recurrent syncope) |
| Phosphodiesterase type-5 inhibitors | **I.5** Phosphodiesterase type-5 inhibitors (e.g., avanafil, sildenafil, tadalafil, vardenafil) for  persistent erectile dysfunction that causes distress | **B.14** Phosphodiesterase type-5 inhibitors (e.g., sildenafil, tadalafil, vardenafil) in severe heart failure characterised by hypotension i.e., systolic BP < 90 mmHg, or concurrent nitrate therapy for angina (risk of cardiovascular collapse) |
| Non-TCA antidepressants | **D2.** Non-TCA antidepressant drug for major depression. | **K8.** Antidepressants in patients with recurrent falls (may impair sensorium). |
| Long-acting muscarinic antagonist (LAMA e.g., tiotropium, aclidinium, umeclidinium, glycopyrronium) or long-acting beta 2 agonist | **G1.** Long-acting muscarinic antagonist (LAMA e.g., tiotropium, aclidinium, umeclidinium, glycopyrronium) or long-acting beta 2 agonist (LABA e.g., bambuterol, formoterol, indacaterol, olodaterol, salmeterol) for symptomatic COPD of GOLD 1 or 2 severity and chronic asthma. | **G3.** Long-acting muscarinic antagonists (e.g. tiotropium, aclidinium, umeclidinium, glycopyrronium) with a history of narrow angle glaucoma (may exacerbate glaucoma) or bladder outflow obstruction (may cause urinary retention). |
