# Supplementary material for: The hidden risks of polypharmacy: Exploring potentially inappropriate prescribing with STOPP/START criteria version 3–A cross-sectional study
Source: PLoS One. 2025 Dec 18;20(12):e0337586. doi: 10.1371/journal.pone.0337586 (PMC12714279; doi:10.1371/journal.pone.0337586)
Supplement: S3 Table — (DOCX) [file pone.0337586.s003.docx]

Distribution of nominal variables including in univariate logistic regressions regarding potentially inappropriate medications (PIM)

|  |  | Presence of cognitive impairment | |
| --- | --- | --- | --- |
|  |  | + | - |
| Presence of at least one PIM | + | 76 | 142 |
|  | - | 24 | 54 |

|  |  | Sex | |
| --- | --- | --- | --- |
|  |  | Male | Female |
| Presence of at least one PIM | + | 70 | 148 |
|  | - | 30 | 48 |

|  |  | Presence of hypertension | |
| --- | --- | --- | --- |
|  |  | + | - |
| Presence of at least one PIM | + | 189 | 29 |
|  | - | 69 | 9 |

|  |  | Presence of heart failure | |
| --- | --- | --- | --- |
|  |  | + | - |
| Presence of at least one PIM | + | 46 | 172 |
|  | - | 20 | 58 |

|  |  | Presence of coronary artery disease | |
| --- | --- | --- | --- |
|  |  | + | - |
| Presence of at least one PIM | + | 64 | 154 |
|  | - | 24 | 54 |
|  |  | Presence of anemia | |
|  |  | + | - |
| Presence of at least one PIM | + | 31 | 187 |
|  | - | 16 | 62 |

|  |  | Presence of diabetes mellitus type 2 | |
| --- | --- | --- | --- |
|  |  | + | - |
| Presence of at least one PIM | + | 79 | 139 |
|  | - | 30 | 48 |

|  |  | Presence of atrial fibrillation | |
| --- | --- | --- | --- |
|  |  | + | - |
| Presence of at least one PIM | + | 45 | 173 |
|  | - | 23 | 55 |

|  |  | Presence of benign prostate hyperplasia | |
| --- | --- | --- | --- |
|  |  | + | - |
| Presence of at least one PIM | + | 25 | 193 |
|  | - | 14 | 64 |

|  |  | Presence of urinary incontinence | |
| --- | --- | --- | --- |
|  |  | + | - |
| Presence of at least one PIM | + | 107 | 111 |
|  | - | 30 | 48 |

|  |  | Presence of ischemic stroke | |
| --- | --- | --- | --- |
|  |  | + | - |
| Presence of at least one PIM | + | 58 | 160 |
|  | - | 20 | 58 |

|  |  | Presence of depression | |
| --- | --- | --- | --- |
|  |  | + | - |
| Presence of at least one PIM | + | 58 | 160 |
|  | - | 12 | 66 |

|  |  | Presence of recurrent falls | |
| --- | --- | --- | --- |
|  |  | + | - |
| Presence of at least one PIM | + | 49 | 169 |
|  | - | 8 | 70 |

|  |  | Presence of sleep disturbances | |
| --- | --- | --- | --- |
|  |  | + | - |
| Presence of at least one PIM | + | 31 | 187 |
|  | - | 12 | 66 |

|  |  | Presence of constipation | |
| --- | --- | --- | --- |
|  |  | + | - |
| Presence of at least one PIM | + | 64 | 154 |
|  | - | 20 | 58 |

|  |  | Presence of osteoarthritis | |
| --- | --- | --- | --- |
|  |  | + | - |
| Presence of at least one PIM | + | 153 | 65 |
|  | - | 52 | 26 |

Distribution of nominal variables including in univariate logistic regressions regarding potentially prescribing omission (PPO)

|  |  | Presence of cognitive impairment | |
| --- | --- | --- | --- |
|  |  | + | - |
| Presence of at least one PPO | + | 78 | 155 |
|  | - | 22 | 41 |

|  |  | Sex | |
| --- | --- | --- | --- |
|  |  | Male | Female |
| Presence of at least one PPO | + | 79 | 154 |
|  | - | 21 | 42 |

|  |  | Presence of hypertension | |
| --- | --- | --- | --- |
|  |  | + | - |
| Presence of at least one PPO | + | 204 | 29 |
|  | - | 54 | 9 |

|  |  | Presence of heart failure | |
| --- | --- | --- | --- |
|  |  | + | - |
| Presence of at least one PPO | + | 65 | 168 |
|  | - | 1 | 62 |

|  |  | Presence of coronary artery disease | |
| --- | --- | --- | --- |
|  |  | + | - |
| Presence of at least one PPO | + | 84 | 149 |
|  | - | 4 | 59 |

|  |  | Presence of anemia | |
| --- | --- | --- | --- |
|  |  | + | - |
| Presence of at least one PPO | + | 39 | 194 |
|  | - | 8 | 55 |

|  |  | Presence of diabetes mellitus type 2 | |
| --- | --- | --- | --- |
|  |  | + | - |
| Presence of at least one PPO | + | 89 | 144 |
|  | - | 20 | 43 |

|  |  | Presence of atrial fibrillation | |
| --- | --- | --- | --- |
|  |  | + | - |
| Presence of at least one PPO | + | 59 | 174 |
|  | - | 9 | 54 |

|  |  | Presence of ischemic stroke | |
| --- | --- | --- | --- |
|  |  | + | - |
| Presence of at least one PPO | + | 64 | 169 |
|  | - | 14 | 49 |

|  |  | Presence of urinary incontinence | |
| --- | --- | --- | --- |
|  |  | + | - |
| Presence of at least one PPO | + | 112 | 121 |
|  | - | 25 | 38 |

|  |  | Presence of benign prostate hyperplasia | |
| --- | --- | --- | --- |
|  |  | + | - |
| Presence of at least one PPO | + | 37 | 196 |
|  | - | 2 | 61 |

|  |  | Presence of depression | |
| --- | --- | --- | --- |
|  |  | + | - |
| Presence of at least one PPO | + | 60 | 173 |
|  | - | 10 | 53 |

|  |  | Presence of recurrent falls | |
| --- | --- | --- | --- |
|  |  | + | - |
| Presence of at least one PPO | + | 48 | 185 |
|  | - | 9 | 54 |

|  |  | Presence of osteoarthritis | |
| --- | --- | --- | --- |
|  |  | + | - |
| Presence of at least one PPO | + | 164 | 69 |
|  | - | 41 | 22 |

|  |  | Presence of sleep disturbances | |
| --- | --- | --- | --- |
|  |  | + | - |
| Presence of at least one PPO | + | 30 | 203 |
|  | - | 13 | 50 |
